# Construction of BPQDs/Ti_3_C_2_@TiO_2_ Composites with Favorable Charge Transfer Channels for Enhanced Photocatalytic Activity under Visible Light Irradiation

**DOI:** 10.3390/nano10030452

**Published:** 2020-03-03

**Authors:** Ziyu Yao, Huajun Sun, Huiting Sui, Xiaofang Liu

**Affiliations:** 1 State Key Laboratory of Silicate Materials for Architectures, Wuhan University of Technology, Wuhan 430070, China; silicate@whut.edu.cn (Z.Y.); webmaster@whut.edu.cn (H.S.); 2 School of Materials Science and Engineering, Wuhan University of Technology, Wuhan 430070, China; 3 Advanced Ceramics Institute of Zibo New & High-Tech Industrial Development Zone, Zibo 255000, China; 4 School of Chemistry, Chemical Engineering and Life Sciences, Wuhan University of Technology, Wuhan 430070, China

**Keywords:** double heterojunction, photocatalytic degradation, hydrogen evolution reaction, charge transfer

## Abstract

Design and construction of double heterojunction is favorable to improve the separation and migration efficiency of photogenerated carriers, thus preferably solve the problems of environmental pollution and energy crisis. Herein, TiO_2_ nanoparticles (NPs) are in-situ grown on highly conductive Ti_3_C_2_ nanosheets via low-temperature hydrothermal strategy, and then black phosphorus quantum dots (BPQDs) are introduced on the surface of TiO_2_ NPs. Under hydrothermal temperature 120 °C, the BPQDs/Ti_3_C_2_@TiO_2_ photocatalyst exhibits remarkable enhanced photocatalytic degradation of methyl orange (MO) and hydrogen evolution reaction (HER) compared with BPQDs/Ti_3_C_2_ and Ti_3_C_2_@TiO_2_ composites. Enhanced photocatalytic activity can be attributed to (i) the BPQDs with tunable bandgaps are deposited on the TiO_2_ NPs to form intimate heterojunction, which facilitates the electrons transfer from the conduction band (CB) of BPQDs to the CB of TiO_2_; (ii) the electrons quickly migrate from CB of TiO_2_ NPs to the Ti_3_C_2_ nanosheets with excellent electronic conductivity via electron transfer channel, which is beneficial to prolong the lifetime of electrons and hinder the recombination of photogenerated carriers; (iii) the enhanced visible light absorption and enlarged specific surface area of BPQDs/Ti_3_C_2_@TiO_2_ further accelerate the photocatalytic reaction. This work emphasizes the essential role of quantum dots in the construction of double heterojunction and the potential application of Ti_3_C_2_ MXene for improving photocatalytic activity.

## 1. Introduction

Over the past few years, the solution of environmental pollution and energy crisis is inseparable from the progress and development of photocatalytic technology [1,2]. Since Fujishima discovered the phenomenon of water decomposition in 1972, titanium dioxide (TiO_2_) has become the most widely used semiconductor photocatalyst with non-toxicity, low cost, and strong stability [3,4]. Nowadays, the rapid recombination of photogenerated electrons and holes is the primary factor limiting the application of TiO_2_ [5]. Researchers increase the separation time of photogenerated carriers by changing the band gap of TiO_2_ [6,7,8]. In addition, the establishment of heterojunction can also effectively hinder the recombination of photocarriers, thus improving photocatalytic activity [9,10,11,12,13,14].

Recently, the TiO_2_ coupled with two-dimensional (2D) layered materials has been proven to effectively prolong the separation time of photogenerated electrons and holes [15]. The excellent electrochemical performance, outstanding chemical stability, and numerous hydrophilic surface functionalities of two-dimensional (2D) layered MXene can be obtained from MAX phase, which have attracted more and more attention since it was discovered by Barsoum et al. in 2011 [16,17,18,19,20]. The heterojunction of 2D Ti_3_C_2_ MXene can be constructed by strong interface contact between layered structure and semiconductors [21,22,23]. The strong physical and electronic coupling between heterojunctions greatly promotes the separation and migration of photogenerated electron-hole pairs, reducing the recombination efficiency of holes and electrons, thus effectively enhancing the photocatalytic performance [24,25].

As reported, 2D Ti_3_C_2_ MXene layered materials exhibit amazing effects on promoting photocatalytic performance of CO_2_ reduction reaction, hydrogen production, and organic compounds degradation [26,27]. For example, Ti_3_C_2_-OH/P25 composites exhibit excellent photocatalytic activity of CO_2_ reduction, which can be attributed to the effective charge-carrier separation [28]. The TiO_2_/Ti_3_C_2_ MXene composite has synthesized successfully by Yu et al. through calcination method, confirming the excellent photocatalytic CO_2_ reduction performance of TiO_2_ in-situ growth on Ti_3_C_2_ MXene surface [29]. Superior conductivity and high efficiency of charge separation and transfer greatly enhance the hydrogen production performance of the g-C_3_N_4_/Ti_3_C_2_ samples [30]. Whereas, the structure of Ti_3_C_2_ MXene can be destroyed under overtop reaction temperature and transformed into TiO_2_ [29]. Therefore, the structure stability of Ti_3_C_2_ tends to be increased under lower reaction temperature, which enables Ti_3_C_2_ play a better role in electron transfer of photocatalytic reaction.

Especially, the Ti_3_C_2_@TiO_2_@MoS_2_ composite shows remarkable enhancement in the photocatalytic H_2_ reaction, which is attributed to the construction of dual-carrier-separation heterojunction [31]. The In_2_S_3_/anatase TiO_2_@metallic Ti_3_C_2_T_x_ MXene hybrids reveal significantly enhanced photocatalytic degradation of MO due to the build type-II heterojunction and Schottky junction with favorable charge transfer [32]. It can be noted from the previous results that Ti_3_C_2_-based composites with double heterojunction is favorable for the separation and transfer efficiency of photogenerated carriers [31,32,33]. However, it is difficult to establish a perfect contact interface between large-scale materials, thus increasing the barrier of electron transfer at the interface [34]. There is an urgent need to find small-size materials to overcome these problems.

Zero-dimensional black phosphorus quantum dots (BPQDs) with unique photophysical and electrochemical properties can be prepared by liquid exfoliation method from bulk black phosphorus (BP) [35]. The good stability, strong light absorption, and high photoluminescence quantum yields of BPQDs have been utilized as fluorescent probes, lithium storage, and organic photovoltaics (OPVs) [36,37,38]. BPQDs have the characteristics of tunable bandgaps, which can be extended to nearly 3 eV, making it a promising candidate in the visible-light-responsive photocatalysts [39]. Moreover, contact interface is more easily constructed by small-size quantum dots than large-size materials in heterojunction system, which is beneficial for the transfer of photogenerated electrons and enhancement of the visible light absorption [34].

In this work, novel BPQDs/Ti_3_C_2_@TiO_2_ photocatalysts are prepared by low-temperature hydrothermal reaction. The BPQDs/Ti_3_C_2_@TiO_2_ samples exhibit significant enhancement on photocatalytic degradation and photocatalytic hydrogen evolution reaction under visible light irradiation. The structure, morphology, surface chemical state, optical and electrochemical properties are revealed. Especially, the double heterojunction structure is constructed among BPQDs, anatase TiO_2_ nanoparticles, and Ti_3_C_2_ nanosheets, promoting the rapid transfer of electrons in the charge transfer channels, which is beneficial to the significant enhancement of photocatalytic activity. Finally, the possible enhanced photocatalytic performance mechanism is proposed based on the experimental results, which demonstrates that BPQDs and Ti_3_C_2_@TiO_2_ composites have potentially applied in photocatalytic systems.

## 2. Materials and Methods 

### 2.1. Materials

Ti_3_AlC_2_ MAX powders (>98 wt % purity) are purchased by Shanghai Yuehuan Co., Ltd. (Shanghai, China). Hydrofluoric acid, ethanol, and methylene orange (MO) are purchased from Guoyao Chemical Co., Ltd. (Shanghai, China). All the reagents used are of analytical grade and used without further purification.

### 2.2. Synthesis of BPQDs/Ti_3_C_2_@TiO_2_ Composites

**Figure 1:**
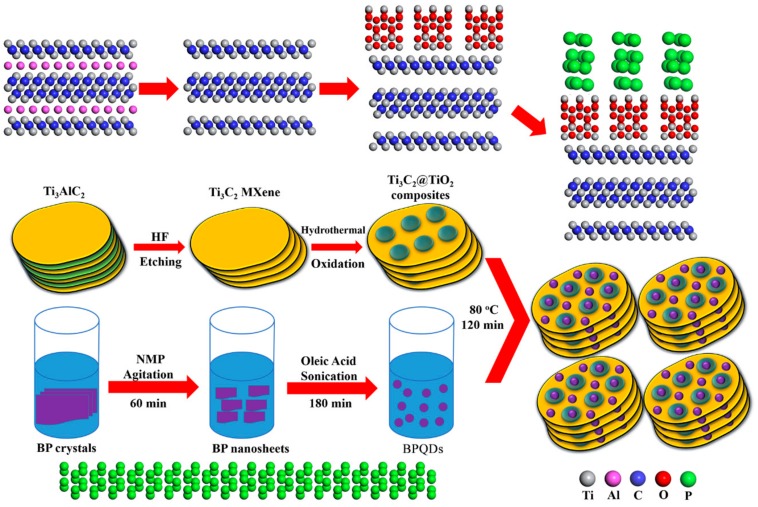
Schematic illustration of the preparation of BPQDs/Ti_3_C_2_@TiO_2_ composites.

Preparing strategy of the BPQDs/Ti_3_C_2_@TiO_2_ (BTTC) composites is illustrated in Figure 1. Firstly, Al layer is completely removed by etching Ti_3_AlC_2_ MAX with 49% HF for 25 h, which is labeled as Solution A. Subsequently, the black powders of Ti_3_C_2_ sample is obtained from Solution A with washing, sonication, and centrifugation processes. Finally, the in-situ growth of TiO_2_ nanoparticles from Ti_3_C_2_ MXene are obtained by hydrothermal reaction with absolute alcohol at different temperature for 16 h, and the Ti_3_C_2_@TiO_2_ composites are synthesized successfully [40]. The corresponding precipitation Ti_3_C_2_@TiO_2_ samples are collected and named as TC (without hydrothermal process) and TTC-x (x = 100, 120, 140, 160 °C).

The BPQDs are prepared by a liquid exfoliation method. In brief, 20 mg of black phosphorus powders is ground for 10 min. The obtained black phosphorus sample is dispersed in 40 mL mixture solution of dimethyl pyrrolidone and oleic acid by strong ultrasound at 0 °C for 4 h. Then, suspension is centrifuged by 11,000 rpm for 30 min to remove the unexfoliated residue. Finally, the supernatant of 0.5 mg mL^−1^ BPQDs is obtained.

The BPQDs/Ti_3_C_2_@TiO_2_ photocatalysts are prepared by solvent-heat method. 50mg of TTC-x (x = 100, 120, 140, 160 °C) sample is added to 50 mL BPQDs supernatant and heated in water bath at 80 °C for 2 h. After the reaction is completed, the precipitate is separated by centrifugation and washed with absolute ethanol. The obtained samples are denoted as BTC and BTTC-x (x = 100, 120, 140, 160 °C).

### 2.3. Photocatalytic Degradation Reaction

The photocatalytic activity of BPQDs/Ti_3_C_2_@TiO_2_ (BTTC-x) and Ti_3_C_2_@TiO_2_ (TTC-x) samples are assessed by the degradation of methylene orange (MO) solution under visible light irradiation. The photocatalytic degradation tests for MO photodegradation are performed by using a 400 W metal halide lamp (the average light intensity is 80 mW cm^−2^, Philips RVP350). In a typical photocatalytic degradation of MO solution, 50 mg of BTTC-x black powders is dispersed into 50 mL MO aqueous solution (10 mg/L). The dispersion solution is treated in the dark for 0.5 h under strong stirring to achieve an adsorption–desorption equilibrium. About 3.5 mL of dispersion solution is taken under certain time intervals and centrifuged for 3 min to remove the photocatalyst. Finally, the concentration of centrifuged MO solution is analyzed at 463 nm by UV-visible specrtrophotometer. The *C*_0_ denotes the initial concentration of MO solution, and *C_t_* denotes the concentration of MO solution at a certain time. The relative absorbance intensity of *C_t_*/*C*_0_ recorded the concentration change of MO solution and embodied the photocatalytic efficiency of the samples.

### 2.4. Photocatalytic Hydrogen Evolution Reaction

The photocatalytic H_2_ evolution tests are performed in a 50 mL quartz flask. 50 mg of photocatalysts are dispersed ultrasonically into 40 mL aqueous solution containing 25% acetone (triethanolamine as sacrificial reagent). Before irradiation, gas (N_2_) is continuously passed through for 35 min to remove the air. The light is provided by a 300 W Xe lamp equipped with a 420 nm cutoff filter to provide the visible light irradiation. The production of H_2_ is detected by gas chromatography (Model BUCK 910, Shanghai Zhao Ming, Shanghai, China) equipped with thermal conductivity detector (TCD).

### 2.5. Characterizations

The crystalline phase of the as-prepared samples is analyzed at 40 kV by X-ray diffractometer (XRD, Cu Kα, Bruker D8 Advance, Karlsruhe, Germany) in the range of 2*θ* = 5°–70°. The morphologies of the BTTC-x samples are obtained by field emission scanning electron microscopy (FESEM, Zeiss Ultra Plus, Carl *Zeiss*, Oberhausen, Germany) coupled with energy-dispersive spectrometry (EDS). The heterojunctions between BPQDs and Ti_3_C_2_@TiO_2_ are performed by high resolution transmission electron microscopy (HRTEM, JEM-2100F, Japanese electronics, Tokyo, *Japan*). The optical properties and UV-vis absorption spectra of photocatalysts are performed by using a UV-vis diffuse reflectance spectroscope (DRS, Lambda 750S, PerkinElmer, New York, NY, the United States) with an integrated sphere. The BET specific surface area (SSA) and pore volume are carried out by using a Micromeritics ASAP 2460 system at 77 K. The photocurrent measurements and electrochemical spectra are measured by 1030 A CHI electrochemical station, which is consisted of glassy carbon electrode (GCE), platinum sheets, silver-silver chloride (Ag/AgCl) is saturated KCl as working electrode, counter electrode, and reference electrode, respectively. 30 mL of 1 M HCl solution is utilized as electrolyte. In a typical test, 5.0 mg of catalysts and 110 μL of 5 wt% Nafion solution are dispersed in 2.5 mL of 1:1 v/v ethanol and water with 9 min sonication to form homogeneous suspension. Prior to the modification, the GCE is polished with alumina powder (1.0 and 0.5 μm), being cleaned with ultrapure water, and then dried in air. Subsequently, 10 μL of the ink is dropped onto the GCE surface. The electrochemical measurements are carried out with the same configuration at overpotential n = 200 mV from 0.1 to 100 kHz with an AC voltage of 5 mV.

## 3. Results and Discussion

**Figure 2:**
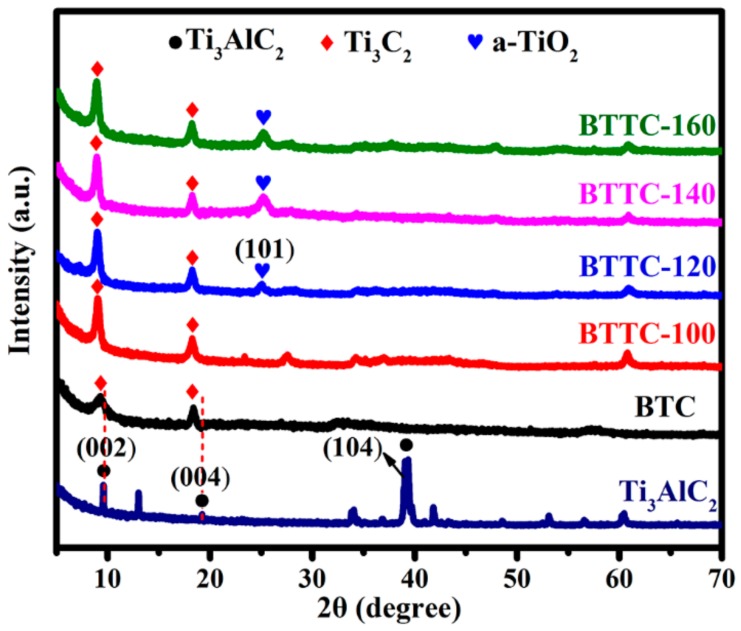
XRD patterns of raw Ti_3_AlC_2_, BTC, and BTTC-x (x = 100, 120, 140, and 160 °C, respectively).

The crystalline of BTC and BTTC-x samples is analyzed in Figure 2. Obviously, the diffraction peak for (104) of Ti_3_AlC_2_ raw sample at 2*θ* = 39° disappears after etching with HF for 25 h. Besides, the diffraction peaks for (002) at 2*θ* = 9.72° and (004) at 2*θ* = 19.18° of Ti_3_AlC_2_ are broadened and shifted to lower angle, suggesting that Ti-Al bond is broken and Ti_3_C_2_ is prepared successfully [41]. The XRD of Ti_3_C_2_ and TTC-x (x = 100, 120, 140, and 160 °C, respectively) is revealed in Figure S1. No obvious diffraction peak of anatase TiO_2_ can be observed, suggesting that this temperature is not conductive to generate anatase TiO_2_. With the increase of the hydrothermal temperature, a new peak at 2*θ* = 25.4° is detected for BTTC-120/140/160 compared with the BTTC-100 sample, which is attributed to the (101) crystal face of anatase TiO_2_ (JCPDS No. 21-1272) [40]. Meanwhile, the intensity of diffraction peak at 2*θ* = 25.4° increases as rising the hydrothermal temperature, which proves that more Ti_3_C_2_ transforms to TiO_2_ NPs. No obvious diffraction peak of BPQDs can be detected, which can be attributed to the low BPQDs-loading in the composites.

**Figure 3:**
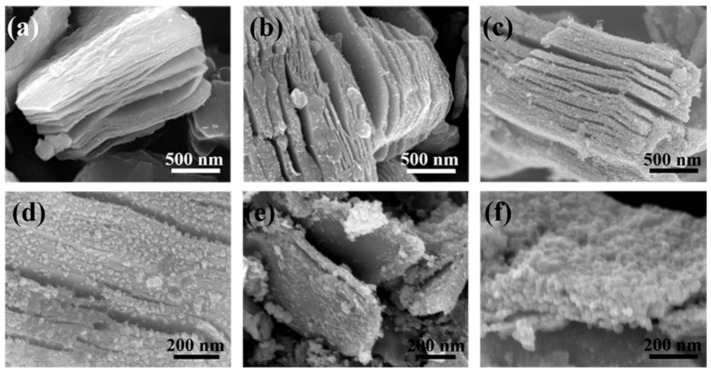
FESEM images of: (**a**) BTC, (**b**) BTTC-100, (**c**,**d**) BTTC-120, (**e**) BTTC-140, and (**f**) BTTC-160.

The FESEM images show the morphologies of BTC and BTTC-x composite (x = 100, 120, 140, and 160 °C) in Figure 3. The typical 2D layered Ti_3_C_2_ with accordion-like structure is obtained in Figure 3a, indicating that the Al layer of raw Ti_3_AlC_2_ is etched by HF solution [42]. Figure 3b shows the accordion-like Ti_3_C_2_, with TiO_2_ nanoparticles attached to its surface. These nanoparticles are arranged on the surface of the sample irregularly with a size of ca. 15 nm, which indicates that the transformation of Ti_3_C_2_ into TiO_2_ NPs can occur after hydrothermal oxidation at 100 °C. The surface of BTTC-120 becomes more rough than raw BTC and BTTC-100, suggesting that more TiO_2_ NPs are formed on the surface of Ti_3_C_2_, as shown in Figure 3c,d. It is noteworthy that TiO_2_ NPs with a size of ca. 25 nm are uniformly dispersed on Ti_3_C_2_ MXene, indicating that the conversion of Ti_3_C_2_ into TiO_2_ can inhibit the agglomeration and promote the uniform size distribution of TiO_2_ NPs at this temperature [29]. Furthermore, the interlayer gap can be modulated with the change in TiO_2_ NPs size, as noted that with the increase of the NPs size, the interlayer gap is broadened [29]. However, further increase of the hydrothermal temperature to 140 °C leads to the destruction of Ti_3_C_2_ nanosheets, which may reduce the electron migration of samples, as revealed in Figure 2e. The size of nanoparticles tend to be increased, and the lamellar structure is seriously destroyed with rising the hydrothermal temperature. Finally, the surface of Ti_3_C_2_ is completely covered by TiO_2_ NPs with the size of ca. 30 nm as the temperature reaches 160 °C, as displayed in Figure 3f. The morphology of BPQDs is hardly observed in the SEM images, which is highly related to the small size.

**Figure 4:**
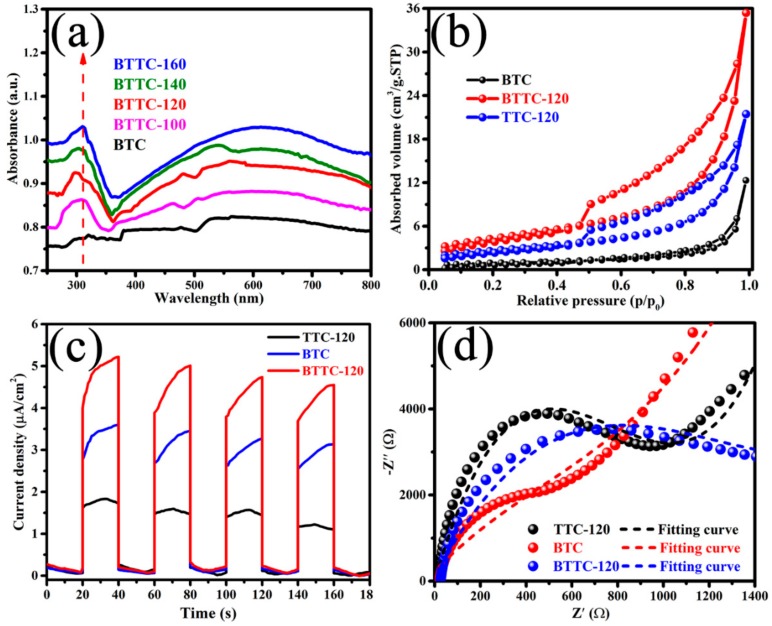
(**a**) UV-vis diffuse reflectance spectra (DRS) of as-synthesized BTC and BTTC-x (x=100, 120, 140, and 160 °C, respectively); (**b**) N_2_ adsorption-desorption isotherms of the as-prepared BTC, TTC-120, and BTTC-120 powders; (**c**) Photocurrent response and (**d**) electrochemical impedance spectra of BTC, TTC-120, and BTTC-120.

Figure 4a gives the DRS spectra of the BTC and BTTC-x samples. Obviously, the prepared BTC reveals the lowest absorption intensity among all the samples, suggesting the sample possesses the worst absorption ability in the range of visible and UV ranges. In comparison, all the BTTC-x samples present better light absorption ability than BTC, indicating that hydrothermal oxidation reaction of Ti_3_C_2_ is beneficial for the enhancement of optical absorption performance. The intrinsic light absorption edge of TiO_2_ at ca. 400 nm can be observed, which is contributed to the emergence of TiO_2_ NPs [29]. Figure S2 reveals the DRS spectrum of BPQDs. The bandgap of BPQDs is estimated to be approximately 2.83 eV.

Figure 4b shows the specific surface areas (SSA) of the BTC, TTC-120, and BTTC-120 powders, which is obtained via the BET analysis. All the samples present typical IV isotherms, proving the mesoporous character of the powders [42]. The BET specific surface areas of BTC, TTC-120, and BTTC-120 samples are 3.1540, 10.5483, and 15.2944 m²/g, respectively. The BTTC-120 composite reveals much larger specific surface area than BTC due to the TiO_2_ NPs in-situ growth on the Ti_3_C_2_ MXene nanosheets [29]. BTTC-120 has larger SSA than TTC-120, due to its composition with BPQDs. The BTTC-120 sample with larger SSA is beneficial for the enhancement of the adsorption and migration of reactants and products.

The separation of electrons and holes of BTC, TTC-120, and BTTC-120 is revealed by transient photocurrent response (TPC), as displayed in Figure 4c. The transient photocurrent of BTTC-120 sample is much higher than BTC and TTC-120, indicating the BPQDs-loading and hydrothermal oxidation can improve the separation of photo-generated carriers on the surface of photocatalysts. In addition, Figure 4d gives the EIS to explore the charge carrier recombination/transfer behavior of BTC, TTC-120, and BTTC-120. The BTTC-120 displays smaller semicircle diameter than TTC-120 under visible light irradiation, indicating the BPQDs can reduce the charge transfer resistance of TTC-120. Additionally, the BTTC-120 exhibits the bigger radius than BTC sample, demonstrating that the sample with higher Ti_3_C_2_ content possesses lowest charge transfer resistance.

**Figure 5:**
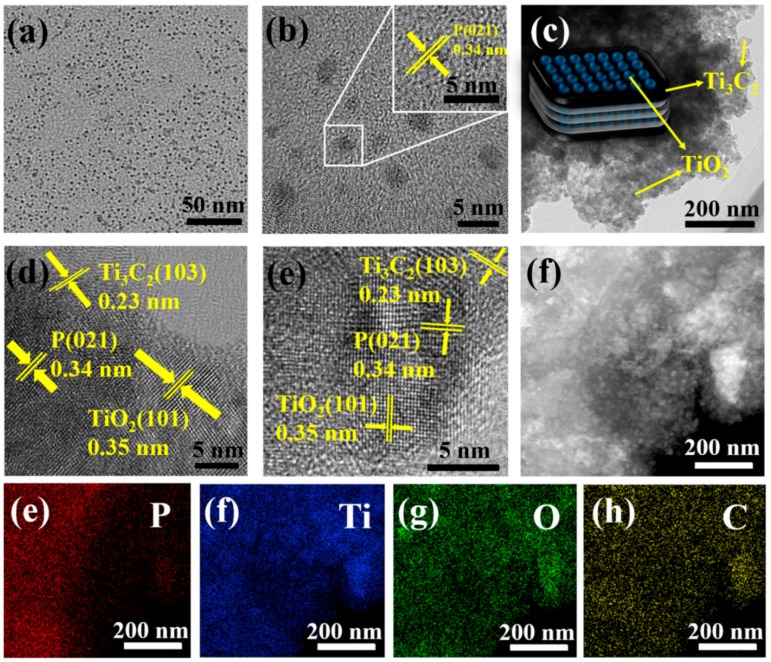
(**a**,**b**) HRTEM images of BPQDs; (**c**–**e**) HRTEM images of BTTC-120 sample; and (**f**–**h**) element mappings of P, Ti, O, and C of BTTC-120.

More morphological details of BPQDs and BTTC-120 samples are further observed in Figure 5. The black phosphorus nanosheets can be observed in Figure S3. Figure 5a,b reveal the TEM images of uniformly dispersed BPQDs with the size distribution between 2.6–4.5 nm. The representative HRTEM image (insets in Figure 5b) of BPQDs displays lattice fringe of 0.34 nm, owing to the (021) planes of BP crystals. Note that the surface of BTTC-120 becomes rough due to the formation of TiO_2_ NPs (Figure 5c), indicating that the TiO_2_ NPs are formed on the Ti_3_C_2_ surface during the hydrothermal process. The TiO_2_ NPs with the size ca. 25 nm present dense features on the surface of Ti_3_C_2_ nanosheets, with characteristics well corresponding to the above FESEM images. As shown in Figure 5d,e, HRTEM images demonstrate the heterojunction formation of BPQDs and TiO_2_ nanoparticles with well-defined lattice fringes on the surface of Ti_3_C_2_. The lattice fringes with an interplanar space of 0.35, 0.23, and 0.34 nm can be clearly observed, which can be ascribed to the (101) planes of anatase TiO_2_, (103) planes of Ti_3_C_2_ nanosheets and (021) planes of BPQDs crystals, respectively [39,40]. It can be confirmed from HRTEM results that the BPQDs have been decorated on the surface of Ti_3_C_2_@TiO_2_ successfully and intimate integration. Furthermore, the heterojunction between BPQDs and Ti_3_C_2_@TiO_2_ has been constructed successfully in BPQDs/Ti_3_C_2_@TiO_2_ photocatalysts. Undoubtfully, the intimate contact heterojunction with strong electronic coupling effect can significantly enhance the charge transfer efficiency at the heterojunction interface [31].

Figure 5e,h present the STEM and EDS mapping results to explore the distribution and composition of element in BTTC-120 sample. The distribution of P is highly overlapped with Ti, O, and C, indicating the composition of BPQDs on TiO_2_ NPs and Ti_3_C_2_ nanosheets. The colorful images exhibit the distribution of P, Ti, O, and C elements, and all the elements are uniformly dispersed in BTTC-120 sample. Above evidence of HRTEM and FESEM images proves that the BTTC-x composites have been synthesized successfully.

**Figure 6:**
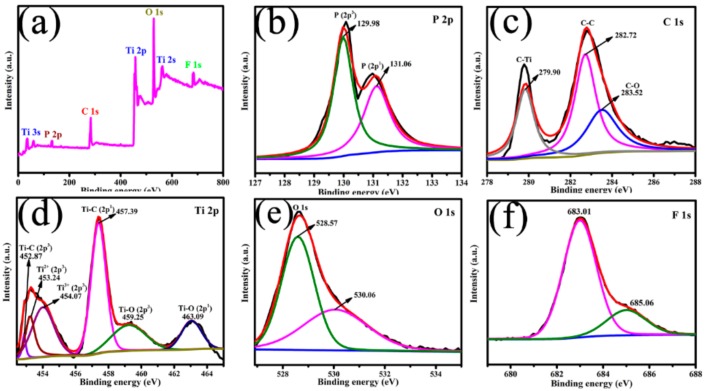
(**a**) XPS survey spectra and high resolution XPS spectra of (**b**) P 2p, (**c**) C 1s, (**d**) Ti 2p, (**e**) O 1s, (**f**) F 1s of BTTC-120 sample.

XPS is utilized to investigate the chemical composition and elements states of BTTC-120 sample, with detailed information as revealed in Figure 6. Figure 6a shows the survey scan of the BTTC-120 composite. The concomitant of P, Ti, C, O, and F elements can be observed in the spectrum, corresponding well to the EDS mapping results. As displayed in Figure 6b, the P 2p spectrum is deconvoluted into two peaks at 129.98 and 131.06 eV, which is ascribed to P (2p^3^) and P (2p^1^), respectively [39]. The C 1s region (Figure 6c) is fitted with three peaks at 279.90, 282.72, and 283.52 eV, being assigned to the Ti–C, C–C, and C–O bonds, respectively [43]. As shown in Figure 6d, peaks at 452.87, 453.24, 454.07, 457.39, 459.25, and 463.09 eV can be attributed to Ti–C (2p^3^), Ti^2+^ (2p^3^), Ti^3+^ (2p^3^), Ti–C (2p^1^), Ti–O (2p^3^), and Ti–O (2p^1^), respectively [44]. In addition, the intensity of Ti–C peak is higher than Ti–O peak, indicating that the Ti_3_C_2_ MXene nanosheets are well preserved after processing via lower-temperature hydrothermal reaction. The O 1s XPS spectrum (Figure 6e) of BTTC-120 is deconvoluted into two peaks at 528.57 and 530.06 eV, which is corresponding to the Ti–O–Ti and surface hydroxyl groups, respectively [45]. In Figure 6f, the F 1s peak located at 686 eV is ascribed to F ions physically adsorbed onto the BTTC-120 surface.

**Figure 7:**
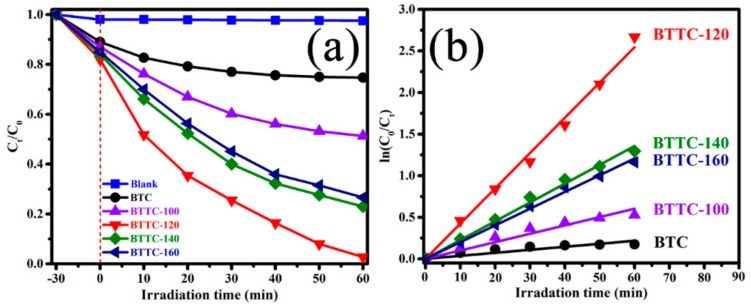
(**a**) Comparison on the photocatalytic efficiency of pristine BTC and BTTC-x composites under visible irradiation (10 mg/L MO (methylene orange) solution); (**b**) the corresponding rate constant k values of BTC and BTTC-x samples.

The photocatalytic activity of BTC and BTTC-x catalysts is investigated by the degradation of MO solution (10 mg/L) under visible irradiation. As revealed in Figure 7a, there is no obvious change for MO solution in the reaction without catalyst, suggesting MO molecules are chemically stable. The adsorption effect of samples has been eliminated by stirring the mixtures for 30 min. The subsequent photocatalytic degradation tests are executed with equilibrium MO concentration as initial concentration after adsorption process. Obviously, the photocatalytic degradation of BTTC-x samples is higher than the pristine BTC due to the TiO_2_ NPs formation in the hydrothermal oxidation, suggesting that TiO_2_ NPs play an essential role in prolonging the separation time of photocarriers in the construction of fast electron transfer channels. With the increasing of reaction temperature, the degradation abilities of BTTC samples exhibit an obvious trend of increase firstly and decreasing after that. Interestingly, the BTTC-120 sample has the highest degradation efficiency among all samples, and more than 93% MO solution is degraded within 60 min. The BTTC-140 and BTTC-160 samples also present better photocatalytic activity, with MO solution degrading by 78% and 76%, respectively. Meanwhile, only nearly 50% of MO is degraded by the BTTC-100 catalyst. This phenomenon indirectly indicates that there is a critical temperature in Ti_3_C_2_ MXene hydrothermal reaction, which can not only in-situ grown anatase TiO_2_ NPs, but also greatly preserves the excellent electronic conductivity of two-dimensional Ti_3_C_2_ MXene, thus preferably enhancing the degradation efficiency of photocatalysts. Figure S4 displays the MO degradation curves over different photocatalysts without BPQDs-loading. The optimal degradation efficiency belongs to TTC-120 sample, 61% MO is degraded in 120 min. Compared with TTC-x samples, all BTTC-x samples possess stronger degradation ability, indicating that BPQDs play a crucial role in the improvement of Ti_3_C_2_@TiO_2_ photocatalytic activity.

In addition, the photocatalytic kinetics of dyes are simulated by the Langmuir–Hinshelwod kinetic theory [46]. The regression curve of natural logarithm normalizes the approximate linearity between MO concentration and reaction time, suggesting that the degradation of MO follows the first-order rate constant [ln(*C*_0_/*C_t_*) = *kt*, where *k* is the apparent first-order rate constant, as revealed in Figure 7b. The kinetics rate constants of BTC and BTTC-x (x = 100, 120, 140 and 160) are 0.01809, 0.05025, 0.21201, 0.11304, and 0.10058 min^−1^, respectively. The enhanced photocatalytic activity of BTTC-x samples is attributed to the intimate contact heterojunction between BPQDs and Ti_3_C_2_@TiO_2_ composites. Different TiO_2_-based composites for photocatalytic degradation of MO under visible light irradiation, as shown in Table 1.

The stability of BTTC-120 photocatalyst is observed by recycling photocatalytic degradation experiment, as shown in Figure S5. The degradation ability of BTTC-120 composite decreases slightly after three degradation recycles, proving that the sample has good stability and sustainability. The structural stability of BTTC-120 is presented by comparing the XRD before and after use, as shown in Figure S6.

**Figure 8:**
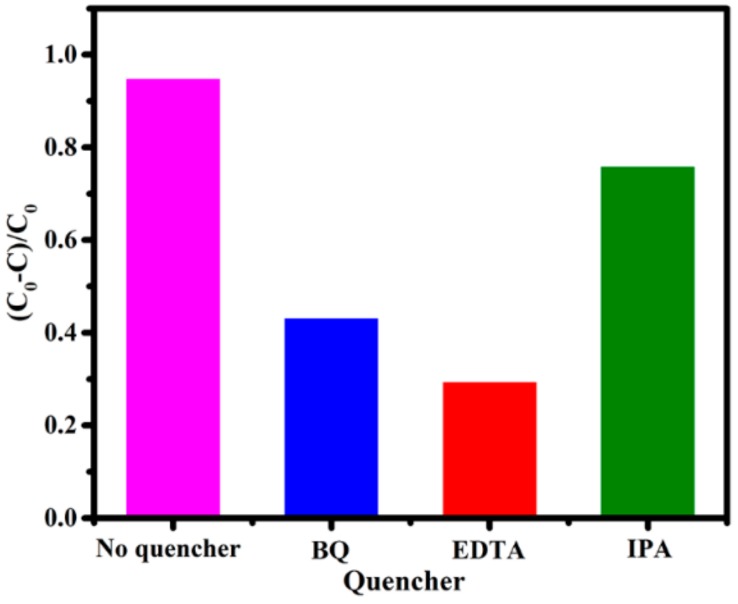
The trapping tests of active species during the photocatalytic degradation of MO over BTTC-120 under visible irradiation.

In order to explore the main active species in the photocatalytic reaction for revealing the photodegradation mechanism. Different trapping agents are added in photocatalytic reaction, as displayed in Figure 8. The addition of IPA (isopropanol, a quencher of •OH) has no obvious effect on the degradation of MO, suggesting that •OH is not the main active species. It can be clearly obtained that MO degradation is obviously inhibited by adding EDTA (triethanolamine, a quencher of h^+^) and BQ (benzoquinone, a quencher of •O_2_^−^), revealing that h^+^ and •O_2_^−^ play important roles in the photocatalytic reaction. It is concluded that the photodegradation of MO over BTTC-120 photocatalyst is driven mainly by the participation of photogenerated holes and •O_2_^−^, and to a lesser extent, by the •OH radicals.

**Figure 9:**
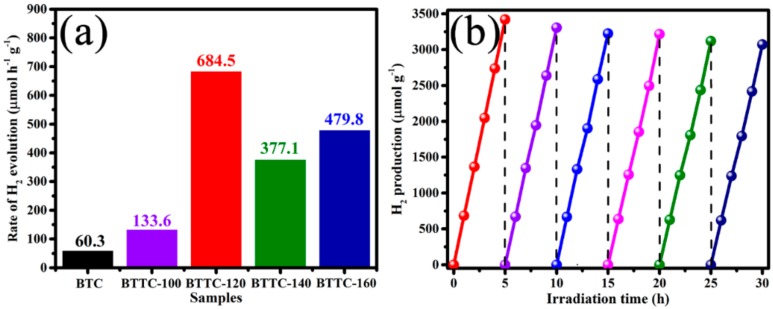
(**a**) The photocatalytic hydrogen evolution rate of BTC and BTTC-x (x = 100, 120, 140, and 160 °C, respectively); (**b**) the recycling tests of BTTC-120 for photocatalytic hydrogen generation process.

The heterojunction of BPQDs/Ti_3_C_2_@TiO_2_ is favorable for the transfer of electrons from CB of BPQDs to Ti_3_C_2_@TiO_2_ composites, thus enhancing the photocatalytic hydrogen rate. Figure S7 and Figure 9a present different hydrogen production rates of TTC-x and BTTC-x with respect to the change in temperature, respectively. Obviously, all the BTTC-x samples exhibit significantly enhanced photocatalytic H_2_ evolution rate than TTC-x composites, indicating that the BPQDs-loading is conductive to the electrons transfer from conduction band of BPQDs to Ti_3_C_2_@TiO_2_ heterojunctions. The photocatalytic H_2_ production rate is enhanced significantly after hydrothermal reaction, suggesting that the emerged TiO_2_ NPs are beneficial to construct a rapid electrons channel between BPQDs and Ti_3_C_2_ nanosheets. The optimal rate of hydrogen production belongs to BTTC-120 (684.5 μmol h^−1^ g^−1^), which is more than two times higher than TTC-120 photocatalyst (324.5 μmol h^−1^ g^−1^), and more than eleven times higher than BTC sample (60.3 μmol h^−1^ g^−1^). The highest H_2_ production rate of BTTC-120 can be attributed to the construction of intimate heterojunction between BPQDs and suitable ratio of anatase TiO_2_ NPs and Ti_3_C_2_ nanosheets in this temperature. The BTTC-100 sample displays a poor hydrogen evolution rate, which is ascribed to the rutile TiO_2_ NPs is not an ideal medium to electrons transfer under low-temperature hydrothermal process. When the temperature rises to 140 °C, the rate of hydrogen production decreases obviously due to the destroyed Ti_3_C_2_ nanosheets. The BTTC-160 shows a higher hydrogen production rate than BTTC-140, which is contributed to the large amount of TiO_2_ NPs generated at higher temperature, thus offsetting the reduced electron transfer due to the destroyed Ti_3_C_2_.

The stability of BTTC-120 sample is evaluated by recycle photocatalytic tests, as shown in Figure 9b. Only a slight loss of hydrogen evolution activity is performed after 6 cycles with 5 h intermittence reaction. It confirms that the strong stability of BTTC-120 photocatalyst is obtained during the photocatalytic experiments.

**Figure 10:**
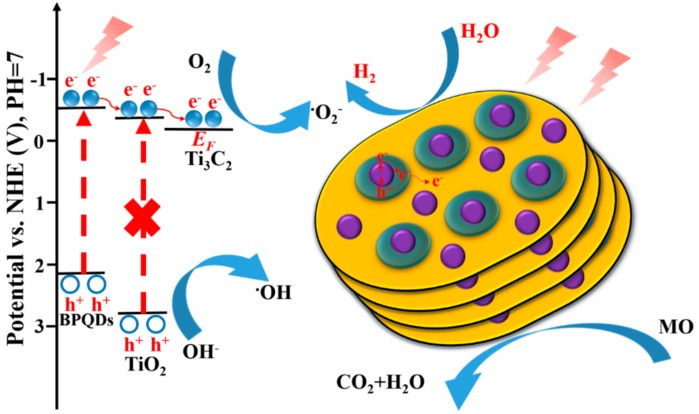
Schematic illustration of photocatalytic reaction of BPQDs/Ti_3_C_2_@TiO_2_ composites under visible irradiation.

Based on the above experiment results, the photocatalytic mechanism of degradation of MO and HER of BPQDs/Ti_3_C_2_@TiO_2_ composites is demonstrated in Figure 10. Firstly, the BPQDs absorbs energy to produce photogenerated electrons and holes in the CB and valance band (VB), respectively. Subsequently, the photoelectrons migrate from CB of BPQDs to the CB of TiO_2_ NPs by intimate contact heterojunction due to the more negative potential [32]. Notably, the Ti_3_C_2_ has more negative Fermi level than the CB of TiO_2_, which is beneficial to the secondary migration of electrons. Photogenerated electrons can easily transfer through the TiO_2_/Ti_3_C_2_ interface with lower energy barrier, thus improving the separation efficiency and prolonging the recombination of photogenerated carriers [31]. On the one hand, a large number of photogenerated holes aggregate on the surface of photocatalyst with powerful oxidation ability, reacting with the adsorbed hydroxyl ions (OH^-^) and water to generate hydroxyl radicals (•OH). On the other hand, the photogenerated electrons aggregate on the surface of Ti_3_C_2_, which reacts with absorbed O_2_ and H_2_O to produce superoxide radicals (•O_2_^−^) to oxidize MO molecules [39]. In the photocatalytic hydrogen production process, the accumulated electrons react with the absorbed H^+^ on the surface of Ti_3_C_2_ to generate H_2_ [30]. The remaining holes at VB are consumed by sacrificial agent, which eliminates the factors affecting the generation of new electron. Eventually, the photocatalytic activity of photocatalytic degradation and hydrogen evolution reaction is enhanced obviously.

## 4. Conclusions

In summary, the novel BPQDs/Ti_3_C_2_@TiO_2_ composites are constructed by in-situ growth of TiO_2_ NPs on the surface of Ti_3_C_2_, and then BPQDs are being introduced onto the TiO_2_ NPs by combining heterojunction nanostructure construction method. The optimal MO degradation efficiency (93%, 60 min) and hydrogen production rate (684.5 μmol h^−1^ g^−1^) belong to BPQDs/Ti_3_C_2_@TiO_2_-120 under visible light irradiation, which is much higher than other BPQDs/Ti_3_C_2_@TiO_2_ and Ti_3_C_2_@TiO_2_ samples. Photogenerated electrons first transfer from the CB of BPQDs with strong visible light absorption to the CB of TiO_2_ NPs, and then transfer to the favorable electrical conductivity of Ti_3_C_2_ MXene due to the existence of surface heterojunction. With the aid of charge transfer channel, it makes the separation and transfer efficiency of photogenerated carriers enhance significantly via the double heterojunction structure. Besides, the enlarged specific surface area of BPQDs/Ti_3_C_2_@TiO_2_ provides enormous adsorption and surface-active sites for photocatalytic process. These results not only demonstrated the promise of Ti_3_C_2_ MXene as an effective photocatalytic material, but also shed light on the crucial role of BPQDs in the photocatalytic degradation and hydrogen evolution reaction.

## Figures and Tables

**Table 1:** Different TiO_2_-based composites for photocatalytic degradation of MO under visible light irradiation.

Composite	Visible Light Source	Catalyst Mass	Dye Concentration	Degradation Efficiency	References
(Fe, N, B)-TiO_2_	1000 W‡tungsten halogen lamp	70 mg	MO, (20 mg/L), 50 mL	300 min, 73%	[47]
Pt-fullerene/TiO_2_	8 W halogen lamp	50 mg	MO, (3.3 mg/L), 50 mL	120 min, 52%	[48]
br-TiO_2_/g-C_3_N_4_	300 W Xe lamp	100 mg	MO, (10 mg/L), 100 mL	180 min, 55%	[49]
TiO_2_-Sn-La	150 W Xe lamp	80 mg	MO, (5 mg/L), 50 mL	120 min, 99%	[50]
PMo12/TiO_2_/Ag	300 W Xe lamp	20 mg	MO, (20 mg/L), 20 mL	120 min, 99%	[51]
L-Histidine (C, N codoped)-TiO_2_-CdS	50 W LED arrays	300 mg	MO, (10 mg/L), 200 mL	120 min, 95%	[52]
In_2_S_3_/anatase TiO_2_ @Ti_3_C_2_T_x_	300 W Xe lamp	60 mg	MO, (20 mg/L), 100 mL	60 min, 90%	[32]
TiO_2_-graphene	450 W Xe lamp	30 mg	MO, (10 mg/L), 50 mL	180 min, 99%	[53]
N-doped rutile TiO_2_	300 W Xe lamp	50 mg	MO, (10 mg/L), 25 mL	120 min, 92%	[54]
BPQDs/Ti_3_C_2_@TiO_2_	400 W metal halide lamp	50 mg	MO, (10 mg/L), 50 mL	60 min, 93%	This work

## Supplementary Materials

The following are available online at https://www.mdpi.com/2079-4991/10/3/452/s1, Figure S1: XRD patterns of Ti_3_C_2_ and TTC-x. (x = 100, 120, 140 and 160 °C, respectively), Figure S2: UV-vis DRS and plots of (*αhv*)^2^ vs *hv* curves of BPQDs, Figure S3: HRTEM image of black phosphorus nanosheets, Figure S4: Comparison on the photocatalytic efficiency of pristine Ti_3_C_2_ and TTC-x composites (10 mg/L MO solution), Figure S5: Cycling degradation curves of MO solution in the presence of BTTC-120 composite, Figure S6: The XRD patterns of used and fresh BTTC-120 sample, Figure S7: The photocatalytic hydrogen evolution rate of Ti_3_C_2_ and TTC-x (x = 100, 120, 140 and 160 °C, respectively).
