# Supplementary material for: Construction of BPQDs/Ti3C2@TiO2 Composites with Favorable Charge Transfer Channels for Enhanced Photocatalytic Activity under Visible Light Irradiation
Source: Nanomaterials (Basel). 2020 Mar 3;10(3):452. doi: 10.3390/nano10030452 (PMC7153474; doi:10.3390/nano10030452)
Supplement: Supplementary file 1 [file nanomaterials-10-00452-s001.pdf]

Supplementary

Construction of BPQDs/Ti_3C_2@TiO_2 Composites with Favorable Charge Transfer Channels for Enhanced Photocatalytic Activity under Visible Light Irradiation

Ziyu Yao [1,2,3], Huajun Sun [1,2,3], Huiting Sui [1,2,3] and Xiaofang Liu [4,*]

[1] State Key Laboratory of Silicate Materials for Architectures, Wuhan University of Technology, Wuhan 430070, China; silicate@whut.edu.cn (Z.Y.); webmaster@whut.edu.cn (Huiting Sui); hsxy@whut.edu.cn (Huajun Sun)

[2] School of Materials Science and Engineering, Wuhan University of Technology, Wuhan 430070, China

[3] Advanced Ceramics Institute of Zibo New & High-Tech Industrial Development Zone, Zibo 255000, China

[4] School of Chemistry, Chemical Engineering and Life Sciences, Wuhan University of Technology, Wuhan 430070, China

* Correspondence: hsxy@whut.edu.cn

Received: 10 January 2020; Accepted: 27 February 2020; Published: date

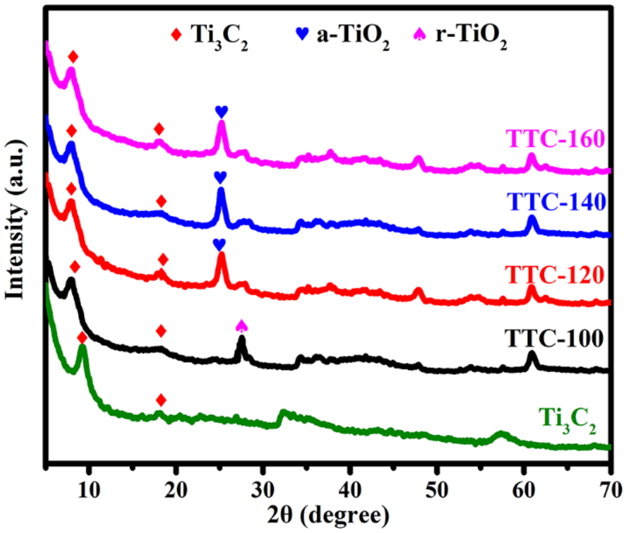

Figure S1: XRD patterns of Ti_3C_2 and TTC-x. (x=100, 120, 140 and 160 °C, respectively).

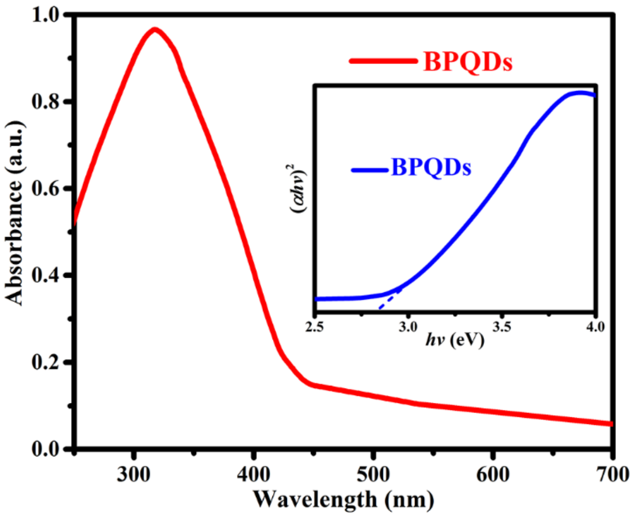

Figure S2: UV-vis DRS and plots of $(\alpha hv)^2$ vs *hv* curves of BPQDs.

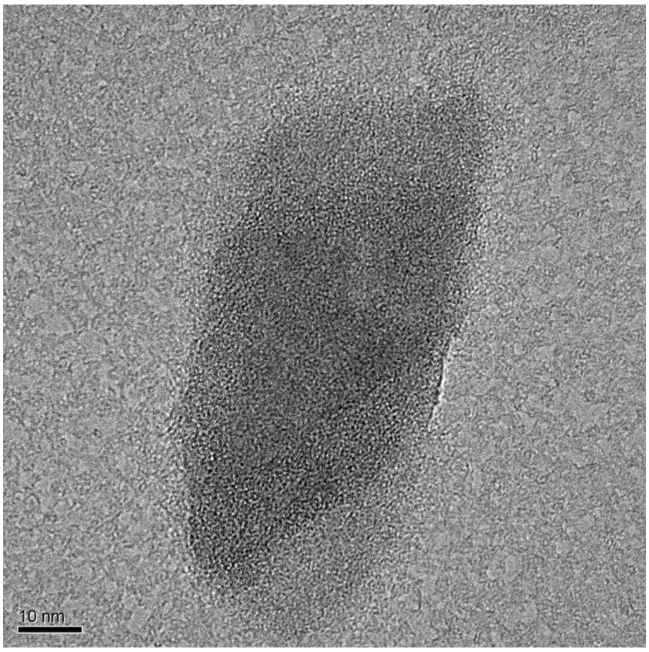

Figure S3: HRTEM image of black phosphorus nanosheets.

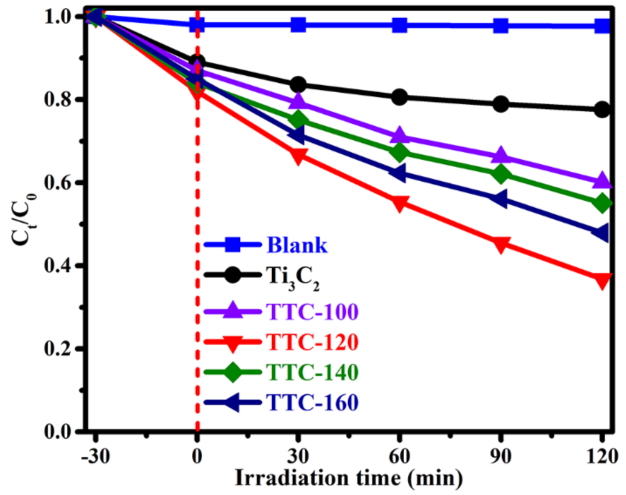

Figure S4: Comparison on the photocatalytic efficiency of pristine Ti_3C_2 and TTC-x composites (10 mg/L MO solution).

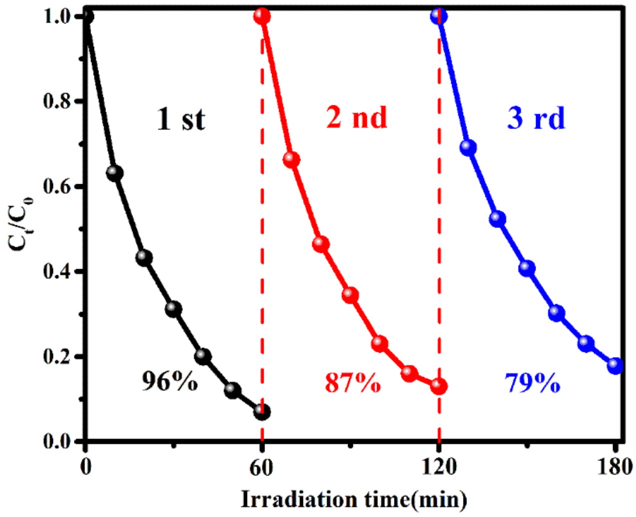

Figure S5: Cycling degradation curves of MO solution in the presence of BTTC-120 composite.

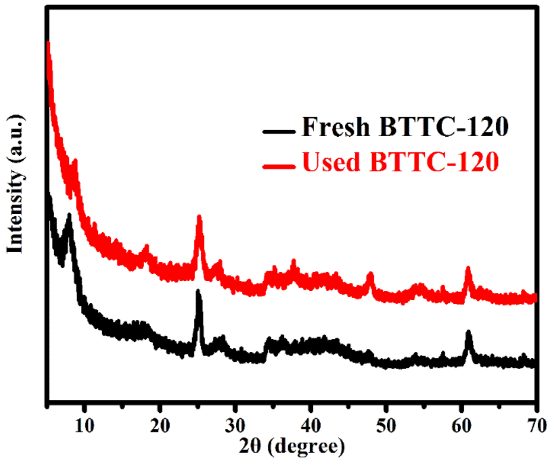

Figure S6: The XRD patterns of used and fresh BTTC-120 sample.

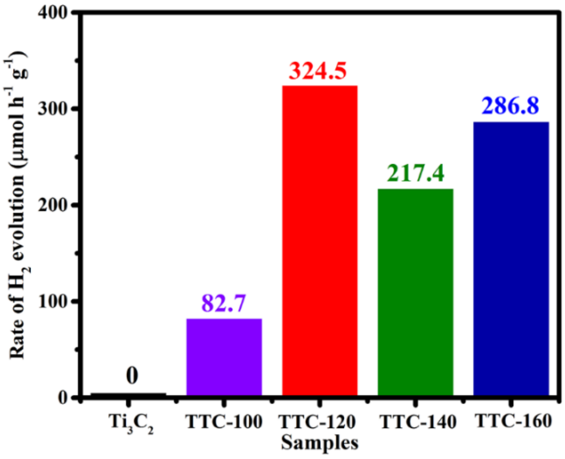

Figure S7: The photocatalytic hydrogen evolution rate of Ti_3C_2 and TTC-x (x=100, 120, 140 and 160 °C, respectively).
